# Applying an equity lens to assess context and implementation in public health and health services research and practice using the PRISM framework

**DOI:** 10.3389/frhs.2023.1139788

**Published:** 2023-04-13

**Authors:** Meredith P. Fort, Spero M. Manson, Russell E. Glasgow

**Affiliations:** ^1^ Centers for American Indian and Alaska Native Health, Colorado School of Public Health, Anschutz Medical Campus, Aurora, CO, United States; ^2^ Department of Health Systems, Management and Policy, Colorado School of Public Health, Anschutz Medical Campus, Aurora, CO, United States; ^3^ Adult and Child Center for Health Outcomes Research and Delivery Science, University of Colorado School of Medicine, Anschutz Medical Campus, and Eastern Colorado Veterans Administration, Aurora, CO, United States

**Keywords:** re-aim, implementation, context, practice, PRISM, representation, reach, health equity

## Abstract

Dissemination and implementation science seeks to enhance the uptake, successful implementation, and sustainment of evidence-based programs and policies. While a focus on health equity is implicit in many efforts to increase access to and coverage of evidence-based programs and policies, most implementation frameworks and models do not explicitly address it. Disparities may in fact be increased by emphasizing high intensity interventions or ease of delivery over meeting need within the population, addressing deep-rooted structural inequities, and adapting to local context and priorities. PRISM (Practical, Robust Implementation and Sustainability Model), the contextual expansion of the RE-AIM (Reach, Effectiveness, Adoption, Implementation, Maintenance) framework has several elements that address health equity, but these have not been explicated, integrated, or illustrated in one place. We present guidance for applying PRISM with an equity lens across its four context domains (external environment; multi-level perspectives on the intervention; characteristics of implementers and intended audience; and the implementation and sustainability infrastructure—as well as the five RE-AIM outcome dimensions. We then present an example with health equity considerations and discuss issues of representation and participation, representativeness and the importance of ongoing, iterative assessment of dynamic context and structural drivers of inequity. We also elaborate on the importance of a continuous process that requires addressing community priorities and responding to capacity and infrastructure needs and changes. We conclude with research and practice recommendations for applying PRISM with an increased emphasis on equity.

## Introduction

There is an urgent need to address health inequities and translation of evidence-based programs into practice and policy. Both goals can be achieved through implementation research and practice efforts—if designed to prioritize health equity and to track and reduce inequities during implementation. However, current approaches may unintentionally increase health disparities. If the underlying multi-level contextual health disparity landscape (historical, political, cultural, economic and social drivers of inequity) and inequitable delivery are not considered in intervention design, adaptation, and uptake, implementation may well perpetuate inequities (1).

RE-AIM is one of the most widely used frameworks for implementation and evaluation research (2). It has been applied with an equity lens in several instances, but usually with limited emphasis on context. Its contextual expansion to PRISM (Practical, Robust Implementation and Sustainability Model) can enhance health equity efforts. The purposes of this paper are to: (1) describe ways that PRISM can be used to support health equity; (2) provide a detailed example of such use; and (3) offer guidance and recommendations for applying an equity lens in future implementation research and practice using the PRISM framework.

## Expansion of RE-AIM to understand external validity and population health impact of programs in context

The purpose of RE-AIM has always been to enhance external validity across diverse settings, including those with limited resources, and public health impact (3). A key enhancement of RE-AIM has been its expansion to the PRISM (4, 5). PRISM adds explicit attention to multi-level contextual factors that impact RE-AIM outcomes. There are four contextual PRISM domains, each of which is multi-level. These are: (1) recipient characteristics (e.g., at citizen, delivery staff, organizational decision makers and community levels); (2) recipient perspectives on the intervention (e.g., history with similar programs, relationships, mental models); (3) external environment (e.g., policies, distribution of resources, health and social system structure and coverage); and (4) implementation and sustainability infrastructure (e.g., resources, and capacity; staff roles and responsibilities; monitoring and evaluation systems).

**Figure 1:**
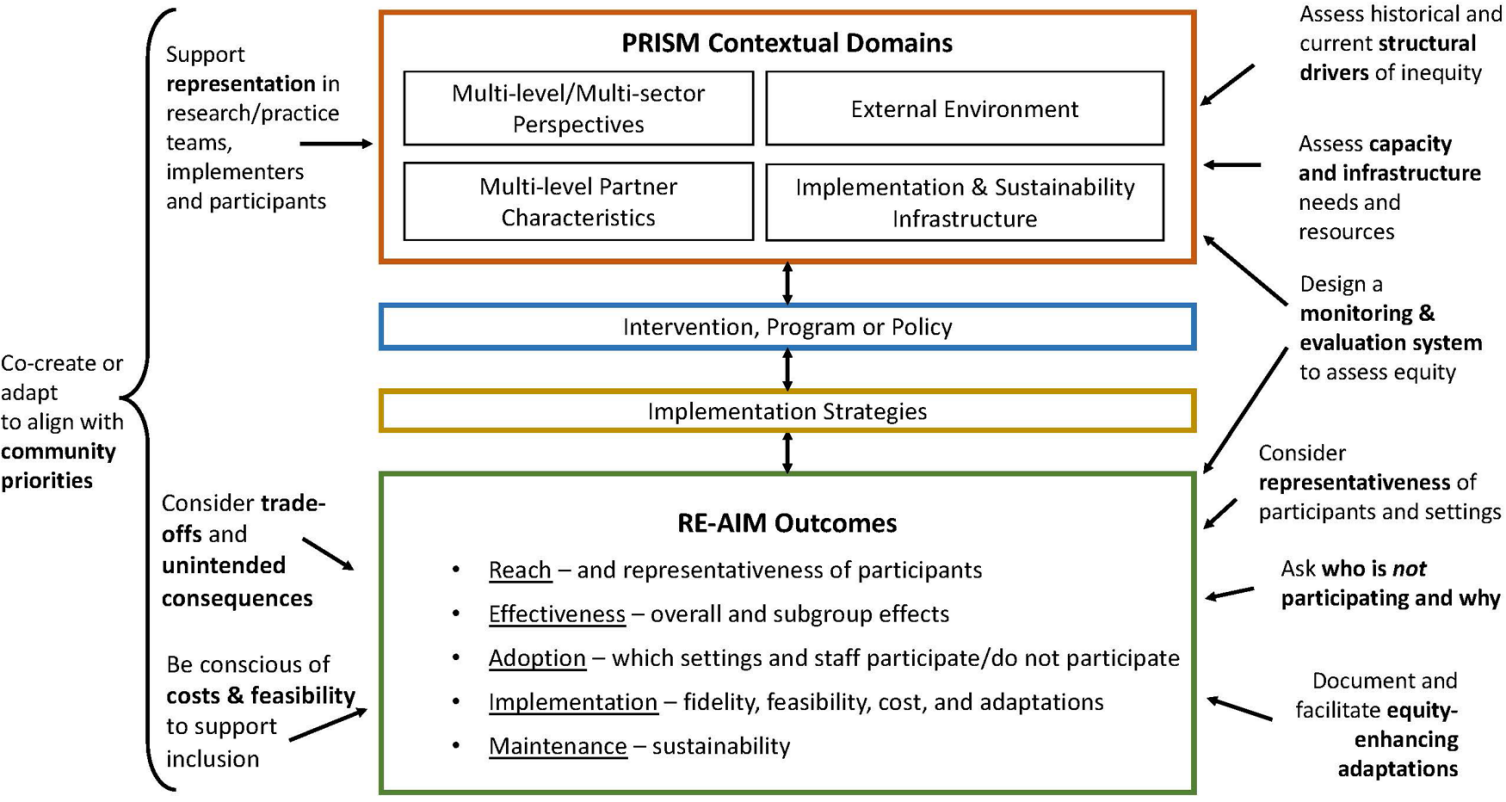
PRISM Contextual Domains and RE-AIM Outcomes with an Equity Lens.

Figure 1 illustrates key features of PRISM as well as examples of actions to enhance health equity. The center column depicts the key PRISM domains and how they interact with the intervention and implementation strategies to deliver the intervention. The combination and alignment of context, the intervention and the implementation strategies produce the RE-AIM outcomes in the lower part of the figure. The example actions summarized on the left- and right-hand side of the figure illustrate how PRISM can be used to enhance equity. Some key opportunities include: (a) attention to representation in planning, implementation and evaluation stages of an intervention; (b) engagement of participants to co-create and/or adapt the intervention and implementation strategies to fit local context and enhance equity; (c) assessment of structural drivers of inequity, and capacity and infrastructure needs and resources; and (d) iterative assessment of RE-AIM outcomes to identify equity-enhancing approaches and address unintended consequences.

Perhaps the most unique contextual factor in PRISM is the implementation and sustainability infrastructure. This component is critical to institutionalizing the assessment of equitable implementation and outcomes. Relevant questions for gauging whether there is adequate infrastructure to assess and promote equity include: Is there staff responsible for tracking equity? Are there reportable equity indicators? This type of equity assessment often defaults to motivated staff or community partners without being not tracked routinely or linked to performance evaluation.

## Working toward equity

In applying an equity lens to implementation research and practice, it is important to consider what aspect of equity a program aims to address (e.g., redistribution of resources to those with the greatest need; equitable participation in the design, implementation and evaluation processes; extension of health care or a social service to a traditionally underrepresented or excluded group, etc.). Programs, policies, or interventions often are stronger in some of these aspects than others. In many cases, equity is not the central focus but rather is considered an outcome to be assessed only after priority assessment of effectiveness—as measured by a clinical health outcome.

Braveman defines health equity as: “the principle underlying a commitment to reduce—and, ultimately, eliminate—disparities in health and in its determinants, including social determinants” (6). Marmot et al. call attention to structural determinants of health that reside outside the health sector (7). This focus reinforces the commitment in the Alma-Ata Declaration to the multisectoral nature of health described as “a world-wide social goal whose realization requires the action of many other social and economic sectors in addition to the health sector” (8) and which is subsequently recognized in the Ottawa Charter for Health Promotion. More recently the Pan American Health Organization has provided guidance for working toward just societies (9). Jones points to the systems of injustice and inequity—racism, sexism, income inequality, and other forms of oppression—that assign value and structure opportunity benefiting some groups more than others (10). Public health and health service fields can improve efforts to address inequities by drawing on the extensive work from other disciplines (e.g., social sciences; political science; public policy and social work).

Within this background, we re-examine PRISM: how it addresses these issues, and present recommendations for how researchers and practitioners can apply the model with an equity lens.

## Context: understanding deep-rooted inequities

Prior to defining the appropriate intervention and adaptations that are needed, it is important to assess the unequal contextual landscape and set goals for health improvement/disparity reduction. In many cases, persistent morbidity and mortality disparities are well-documented and are well-known by communities. However, in implementation research projects, it is common for health and related social and economic disparities to be described almost as a characteristic of the landscape—such as insufficient staffing or lack of access to clean drinking water in a community—rather than the defined problem to be addressed (11).

Underlying drivers of inequity such as colonialism, racism, inequitable access to land, and income inequality are all-too-often viewed as background characteristics and not the focus of change efforts. In some cases, these deep-rooted drivers of inequity are acknowledged, but efforts to address them are targeted at individual-level social needs rather than deeper structural transformation. In PRISM such factors are considered under External Environment or Perspectives of different multi-level participants.

Assessing capacity and implementation and sustainability infrastructure needs in community and health care settings can offer a longer-term road map that links to broader and more sustainable community development and policy change efforts. Inequity in the policy landscape, including the design and structure of health and social service coverage, will influence whether a specific program is offered to different members of the population.

In Table 1, we present definitions of PRISM's contextual factors and RE-AIM outcomes along with a case example applying an equity lens. This project sought to improve hypertension control in Guatemala in intervention districts in rural and indigenous communities in 5 provinces (12, 13). A needs assessment conducted at the outset showed that the health care system, part of the *external environment*, is like many in low- and middle-income countries: the public sub-system requires additional funding and system strengthening to ensure sufficient human resources and medications to adequately meet need across the country (14). Within Guatemala's Ministry of Health, actors at *multiple levels* take part in delivery of the intervention (national-level actors based in the capital, provincial-level Health Areas, and district-level providers), and patients, families, and community members are beneficiaries with important insight about implementation and access. An assessment of explanatory models helped to understand their different perspectives on hypertension (15). *Representativeness* was assessed with census (individual-level) and health administration (setting-level) data. Several districts carried out *equity-enhancing adaptations* during the COVID pandemic to increase patient access to medications by making them available at rural health posts instead of requiring patients to travel to health centers in semi-urban areas; family members were also allowed to pick up medications (16). While some districts had resisted making medications available at rural health posts prior to the pandemic, it was recognized as acceptable during the pandemic. There is an opportunity to build equity assessment into the *implementation and sustainability infrastructure*.

**Table 1:** PRISM contextual factors and RE-AIM outcomes: definitions, equity implications, and an applied example.

PRISM Factors and RE-AIM Outcomes	Details and Examples	Equity implications: illustrative ways to support equitable implementation and to assess factor/outcome with an equity lens	Example from Hypertension Control Project in Guatemala's Ministry of Health System
**Multi-Level Partner Characteristics (citizens, delivery staff, decision makers, community leaders)**	Socio-demographic and other project relevant characteristics at multiple levels and extent of participation by different partners in the design, implementation and analysis	• Consider and co-create ways to achieve equitable participation, especially of traditionally marginalized sectors in the community, in the design, implementation and evaluation • Ensure representation of the beneficiaries’ socio-demographic characteristics among implementers and organizational decision makers • Support establishment and sustainment of multi-sectoral linkages	Implementation research project led by the Institute of Nutrition of Central America and Panama and the Ministry of Health; a cluster-randomized trial with external research funding and collaborating research institutions. **Organizational:** – Experience was focused within the health sector with participation at central, regional, and local levels – Limited representation of Mayan people in decision-making roles within public institutions and among MDs **Beneficiaries/Participants:** – Conducted a pre-implementation engagement process involving patients, family members, providers, and national-level health system stakeholders
**Multi-level Perspectives on the intervention (citizens, delivery staff, decision makers, community leaders)**	History with similar programs, relationships required to deliver the intervention, trust among parties, mental models of intervention effects	• Work over time to build trust, especially among marginalized or socially excluded communities • Create governance structures that allow for different perspectives to be shared in an ongoing way • Elicit and review experiences with similar programs in the past	**Organizational:** – Differing ideas about fit of the intervention/implementation strategies and amount of additional work that is required – Recognition of the importance of primary prevention to curb the tide on surge in non-communicable diseases **Beneficiaries/Participants:** – Conducted an explanatory models of disease analysis capturing perspectives of patients, family members, health care providers and administrators, and national-level health system stakeholders which pointed to: – Use of alternative medicines, beyond biomedical HTN treatment – Challenges of unreliable access to medicines, concerns about cost when HTN medications are not available in the public system
**External Environment**	Policies, guidelines, coverage, level and distribution of resources, current and historical community priorities	• Understand history, legacy and ongoing colonialism, structural racism, and discrimination in policies and norms and work to address them • Influence policy-level and upstream challenges • Support equitable protections/regulations (e.g., environmental protections across communities, restrict advertising that inequitably targets minoritized or low-resource communities, etc.)	– Inequitable society in which rural, Indigenous people experience more social exclusion; history of massacres in rural communities during 36-year internal conflict – Limited public sector budget for health and other social services – Changing food environment; excess availability of low quality, ultra-processed foods and transition away from traditional diets – Dynamic context (e.g., COVID-19 during the implementation period of the trial) that resulted in reduced access to transportation, social distancing rules, and less emphasis on chronic disease programs
**Implementation and Sustainability Infrastructure**	Organizational commitment, resources, and capacity; staff roles and responsibilities; monitoring, evaluation and supervision systems; existence of audit and feedback procedures	• Enable/strengthen data capacity and supervision/evaluation infrastructure to assess equity • Create alignment with organizational mission and priorities • Ensure ongoing program evaluation and assessment of equity impacts is an explicit part of someone's job and performance metrics • Support capacity building/advocate for additional resources in low-resource settings	– Conducted needs assessment applying the WHO's health system building blocks framework—identified capacity building needs and infrastructure gaps including: – Medication availability—need to institute an approach to ensure availability of all three classes of medications for hypertension control in all MOH districts – Need to increase resources for staffing to be able to offer intramural and extramural services in all MOH districts and resources to support supervision of the program (e.g., fuel and transport for site visits, phone minutes for routine check-ins) – Opportunity to develop an equity-focused monitoring and evaluation system with priority indicators for ongoing equity assessment
**RE-AIM Outcomes**			
**Reach (Individual participant level)**	The number, percent and representativeness of individuals who participate vs. those invited/eligible	• Assess characteristics of participants, nonparticipants and those not eligible—capture/measure census-level data for representativeness; apply health equity impact assessment tool • Work with multi-level partners on recruitment strategies, messaging/ interpretation, and locations • Implement strategies to meet underserved/excluded segments of the populations where they are	Assessment of participant representativeness: – Approximately 70% women and 30% men – Work status (most participants do not have formal employment; underrepresentation of employed, especially in agriculture) – Participant identification as Mayan or ladino/a – Literacy/education level – Distance to the health post/center
**Effectiveness (Individual participant level)**	Short-term results on (a) primary outcome; (b) heterogeneity of effects; (c) generalization effects (e.g., quality of life); and (d) unintended consequences	• Assess differences in health outcomes at the outset to determine existing gap • Analyze subgroup effects on outcomes and unintended consequences • Tailor intervention and implementation to individuals; adapt implementation as needed • Include multi-cultural definitions of health outcomes (e.g., wellness; mental + physical + family health)	– Primary effectiveness outcome: BP control – Assessed differences in BP control by patient characteristics – One of the first large-scale efforts to capture and assess data on BP control within the public health system; opportunity to understand and track differences by subgroups to develop strategies for reducing disparities in the future – Important to consider other priority unmet health needs; project focused on HTN control whereas diabetes is more urgent/relevant to many (emphasized in needs assessment)
**Adoption (Multi-level setting and staff levels)**	Number, percent and representativeness of (a) settings (e.g., clinics, communities; organizations) and (b) staff (multiple levels/types of staff)	• Co-create or refine intervention, recruitment and implementation strategies with staff, implementing organization to assure relevance and fit to local context • Promote short- and long-term strategies to increase alignment of setting and staff representativeness with population served • Support capacity building/advocate for additional resources in low-resource settings to enable adoption • Analyze characteristics of participating sites and staff vs. those a) not invited and b) who decline • Assess factors that contribute to limited or slow adoption of the intervention and support the removal of barriers/obstacles and the enhancement of facilitators.	– Representativeness of **staff** (bilingual/ Mayan nurses, primarily Spanish-speaking doctors) – Challenge to fully adopt program and add it to existing workflow, especially with added COVID responsibilities – **Representativeness** of **sites** (selected to have two aux. nurses—most posts have less staff time available; HTN meds were made available for the duration of the study whereas routine availability of medications is not the case in most health posts/centers across the country); conducted assessment of setting-level representativeness using administrative data
**Implementation (Setting and staff levels)**	Fidelity of delivery; adaptions made; and costs of implementation	• Assessment of fidelity and adaptation to guide iterative implementation to increase equity • Recognize the need for differential implementation: offer more to those who need it most; offer less to those who need it least • Understand implementation costs from different perspectives; assess cost to community members, consider investment that could have alternatively been made elsewhere to address underlying causes of inequity	– Identification of **equity-enhancing adaptations** implemented during COVID pandemic (certain flexibilities were supported: 2-month medication supply, allowing family members to pick up meds, offering medications at health post—closer to patients) – Important to assess extent of implementation to those residing in most rural vs. those in more semi-urban communities – Consider family/community support: patients with additional support from family enabled to implement home BP monitoring. – Recognition of the cost to patients (travel, time) to participate (although medications and services are free) – Cost to providers (time, balancing with other responsibilities).
**Maintenance (Individual and Setting levels)**	Longer terms effects of (a) individual outcomes and (b) program delivery at 1–2 or more years	• Analyze % and characteristics of individuals and settings that maintain or adapt program and implementation strategies • Adapt intervention resources required (e.g., task sharing) for sustained implementation • Recognize need for ongoing structural change and improved implementation	– Consider ability for patients to maintain BP control and setting-level sustainability – Medication availability may decline after the project period concludes – Supplies (BP monitors purchased by the project—require maintenance)

## Participation and representation: elevating underrepresented voices

Community-based participatory research defines priorities based on the community's expression of primary concerns and emphasizes representation of those most affected by the focal issue throughout the cycle of problem definition, assessment, interpretation, and dissemination (17). The research or practice problem to be addressed is often structural in nature and requires more than a singular evidence-based program.

In applying PRISM to increase equity, it is important to be aware of and document who has a place at the table and which groups, perspectives, and priorities are included. Equally important is to ask who is *not* at the table and understand why not. It is not sufficient to only engage community members and implementers who are most eager to be involved, have the most time or resources to participate, speak the same language, or share similar backgrounds with the research team. Limited representation in the governance of implementation efforts is likely to perpetuate societal inequities (18). It is especially important to ensure the most marginalized voices are heard rather than default to community leaders or others who may have higher status or access to resources.

Equity of participation across the design, implementation, analysis, and dissemination phases should not be assumed or defined by researchers. Community partners may use tools such as the spidergram developed for assessing community participation (19). Memoranda of understanding and other transparent accountability mechanisms can support communities and partners who come to the table with less power.

Ideally in applying PRISM, community context experts will be involved throughout all phases of a program to improve relevance and prioritization. Community context experts should serve as co-PIs, co-investigators, or in other roles such as community advisory boards. They may identify adaptations of interventions or implementation strategies to render them relevant for their community as well as changes to context to sustain implementation and enhance equity. The next five sections describe equity implications for each RE-AIM outcome.

## Reach: representativeness, generalizability, and structural drivers

RE-AIM focuses attention on who is excluded, who participates or is impacted, who declines or is unable to participate, and the underlying reasons. While RE-AIM has always emphasized representativeness across its five dimensions, under Reach most reports only present data on differences of individual participant characteristics such as age, gender, race, and ethnicity. Typically, individual-level participant characteristics are captured in a “Table 1” with columns that compare those participating in a project and those in a comparison or control group. Comparisons between those who participate and the broader population are rarely reported.

One way to increase equity in Reach, especially in the pre-implementation phase of a project, is to use tools such as the Health Equity Impact Assessment (20). This pre-implementation assessment can identify people from historically-excluded groups, elucidate ways to address barriers to reach, and consider intersectionality, or the multiple, interacting dimensions of inequity at the micro-level that reflect interlocking systems of privilege and oppression at the macro-level (21, 22). Recognizing potential inequities in participation prior to offering a program contrasts with a standard “first come, first served” approach that assumes all individuals have equal ability to participate. Rather than frame low participation as a person-centric issue, implementers should consider it a problem of delivery or design. Programs may need to be delivered in a non-dominant language by staff or peers who share lived experiences with those in the community; over-represented groups may need to be waitlisted to ensure implementation is inclusive and reaches those who have the greatest potential to benefit.

Today there are many efforts to capture social determinants of health and social needs. While important to describe individual-level need, assessments also need to include structural drivers of inequity (23). Focusing data capture on structural drivers forces us to consider additional levels of influence.

As discussed below, representativeness should be assessed across all RE-AIM dimensions. Tools such as the Expanded CONSORT figure can assist with reporting (24) and present an opportunity to understand and document reasons for exclusion and nonparticipation and also recognize capacity building and policy-level needs.

## Effectiveness: expanding assessment beyond individual-level behavioral and clinical primary outcomes

In defining effectiveness outcomes for an intervention, it is important to recognize the assumptions that underlie how health is defined and who determines health improvement metrics. Local knowledge (25) is seldom considered in defining effectiveness outcomes; health benefit is typically operationalized in biomedical terms to address funder or researcher priorities. We should broaden assessments to include measures such as well-being and quality of life and consider different explanatory models of health (26). Western-centric conceptualizations of health often dominate, emphasizing individual-level change, whereas many other cultures view health in broader socio-centric terms of family or community.

We should also capture the heterogeneity of effects and consider whose health improves, whose does not, and why. It is important to assess changes in health outcomes of traditionally marginalized or socially excluded groups. Effectiveness should be evaluated on more than one dimension; for example, an average increase in blood pressure control or daily fruit and vegetable consumption in one dimension, and a reduction in gaps in thesame health outcome measures between groups or neighborhoods at the population level.

## Adoption: setting and staff-level representativeness and capacity building

Sites and communities are often excluded from participating because they lack resources and capacity to meaningfully engage in the process. This may happen explicitly—they are not invited because they do not meet certain criteria—or implicitly—they self-select out in the face of demands of a new evidence-based program. For sites and delivery staff afforded the opportunity to adopt a new program or policy, investing in capacity (human and/or financial resources or physical infrastructure) may be necessary to facilitate adoption. Research and practice may not be able to address long-term capacity needs within their respective lifecycles; however, they may still contribute to equity by identifying the capacity required. A needs assessment conducted prior to implementation can clarify strengths and weaknesses in capacity. Public health and health care system frameworks (27, 28) that examine different system components and capacity domains with a systems strengthening perspective offer ways to identify and prioritize needs.

## Implementation: addressing inequities in delivery; iterative assessment; and prioritizing adaptations that support equity

Programs should design, implement, and adapt evidence-based interventions to local circumstances, recognizing how the inverse care law (29) operates within their context. Fifty years ago, Hart wrote that: “The availability of good medical care tends to vary inversely with the need for it in the population served.” Risk stratification—implementing more care or offering more services or programs for those who have greater health and social care needs—can offset the inverse care law. In some health systems, risk stratification at the family or household level is built within the delivery approach. Such approaches contrast with frequently offering the same intervention to all participants and sites (e.g., the same number of sessions of an evidence-based prevention class) despite differing levels of resources, capacity and need. The targeted or proportionate universalism approach also calls for actions to be implemented with an intensity and a scale proportional to the level of disadvantage (30, 31).

During implementation, PRISM focuses on adaptations to fit local setting resources and changing context. Adapting evidence-based programs or implementation strategies to enhance their delivery in different settings is almost always necessary to fit local culture, history, and resources. Fundamental co-creation and co-design of interventions tailored to community realities is critical and we support the recommendations offered by other colleagues (32). Adaptations during implementation are often needed to improve equity; these equity-enhancing adaptations should be documented and supported (33). Incorporating knowledge from the community experiencing inequities into the program or practice should occur on an ongoing basis and should ideally be built into the implementation and evaluation process. We need to be mindful of potential implementation-generated inequalities, which are more common in some technology-based interventions (34). Monitoring and acting on emerging data through iterative assessment can increase program success and identify equity-enhancing adaptations (35).

## Maintenance and sustainability: enabling long-term implementation and equity assessment

Capacity for sustainability should be assessed to understand the extent to which a setting supports the structures and processes that promote sustained evidence-based programs (36). Too often, low resource settings fund services through undependable grant cycles that compromise sustaining positions and programs (37). Frequently, settings lack the level of staffing or resources to continue a program after conclusion of the active intervention. Thus, it can be helpful to conduct a sustainment or replication cost analysis of the financial impact of different sustainment strategies to help with decision making (38).

## Discussion: research and practice recommendations

Researchers and practitioners can assist efforts to improve equity by documenting context prior to, during, and post-implementation—in each cycle of a program (e.g., including planning, implementation, and evaluation). Ideally, researchers and practitioners should apply an equity lens that simultaneously considers: (1) equity in the implementation process and outcomes (RE-AIM) for a given cycle and (2) the PRISM contextual factors, recognizing that efforts to promote equity on both will be mutually reinforcing. Ongoing contextual insight will identify needed structural change; program implementers can inform and advocate for infrastructure improvement, resource distribution, and policy change to address persistent gaps and societal inequities.

RE-AIM has often been characterized as the product of its dimensions (Reach X Effectiveness X Adoption X Implementation X Maintenance). An important consideration for applying RE-AIM is its implications for equity of trade-offs among different outcomes and potential unintended consequences. Maximizing impact on one dimension may produce adverse impacts on other dimensions. For example, focusing on enhancing intensity of a program may result in reduced adoption by settings and staff. Similarly, it is challenging to capture and equally weight the various PRISM contextual factors. Unanticipated consequences could also be compensatory effects elsewhere (e.g., harm to the environment or future generations) or inadvertent exacerbation of health disparities. Using systems thinking tools and methods such as behavior over time graphs or dynamic modeling to consider different scenarios prior to implementation is one way to build in consideration of unanticipated consequences (39).

An increased emphasis on multi-sectoral interventions and Health in All Policies approaches promise to increase health equity by working to influence social determinants of health. Using PRISM in combination with equity-focused theories, models and frameworks has great potential for advancing health equity.

## Conclusions

This paper adds to the existing literature on health equity and PRISM by: (1) describing equity implications for each PRISM contextual factor and RE-AIM outcome, (2) providing a concrete example to illustrate these issues, and (3) making recommendations for future research and practice. We have not sought to be comprehensive, but rather pragmatic and provide guidance for increasing an equity lens in applying PRISM. We emphasize aspects of equity such as representation, recognizing the potential for unintended consequences that contribute to increasing inequity. It is also important to consider and document changes to the intervention context such as through capacity building and systems level efforts. Finally, we highlight the centrality of the implementation and sustainability infrastructure to enable sustained assessment of equity.

## Data Availability

The original contributions presented in the study are included in the article/Supplementary Material, further inquiries can be directed to the corresponding author.
